# A resegmentation‐shift model for vertebral patterning

**DOI:** 10.1111/joa.12540

**Published:** 2016-09-01

**Authors:** Lizzy Ward, Susan E. Evans, Claudio D. Stern

**Affiliations:** ^1^ Department of Cell and Developmental BiologyUniversity College LondonLondonUK

**Keywords:** carbocyanine dye, centrum, dens, fate map, intervertebral disc, odontoid process, skeletal development

## Abstract

Segmentation of the vertebrate body axis is established in the embryo by formation of somites, which give rise to the axial muscles (myotome) and vertebrae (sclerotome). To allow a muscle to attach to two successive vertebrae, the myotome and sclerotome must be repositioned by half a segment with respect to each other. Two main models have been put forward: ‘resegmentation’ proposes that each half‐sclerotome joins with the half‐sclerotome from the next adjacent somite to form a vertebra containing cells from two successive somites on each side of the midline. The second model postulates that a single vertebra is made from a single somite and that the sclerotome shifts with respect to the myotome. There is conflicting evidence for these models, and the possibility that the mechanism may vary along the vertebral column has not been considered. Here we use DiI and DiO to trace somite contributions to the vertebrae in different axial regions in the chick embryo. We demonstrate that vertebral bodies and neural arches form by resegmentation but that sclerotome cells shift in a region‐specific manner according to their dorsoventral position within a segment. We propose a ‘resegmentation‐shift’ model as the mechanism for amniote vertebral patterning.

## Introduction

A segmented body plan is a conserved feature of the vertebrates. This pattern is set up in the embryo by the segmentation of the paraxial mesoderm into somites, which contain the precursors of the muscles and axial skeleton. Somites form by budding off sequentially from the rostral tip of the presomitic mesoderm (PSM) on either side of the midline, forming an epithelial sphere surrounding a central lumen (‘somitocoel’). Later, the somite differentiates into two sub‐compartments: the dermomyotome, which gives rise to muscles and dermis, and the sclerotome, which contributes to the vertebral column (Christ & Wilting, 1992; Christ & Ordahl, 1995). The sclerotome and dermomyotome initially sit within the same somitic segment. However, for the vertebral column to bend, each axial muscle must insert into two successive vertebrae. Therefore, the sclerotome must shift by half a segment with respect to the dermomyotome during development.

**Figure 1:**
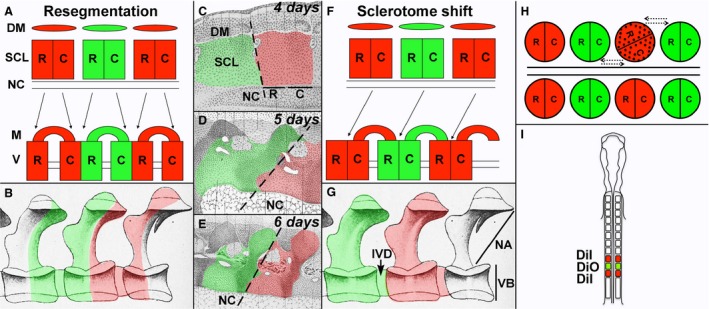
Models of vertebral development. (A) ‘Resegmentation’ model. (B) According to the resegmentation model, a vertebra comprises cells from two consecutive somites (Red and Green; Illustrated vertebrae modified from Lillie, 1936). (C–E) Illustrated oblique sagittal sections through the sclerotome at 4, 5 and 6 days (modified from Schauinsland, 1905; in Lillie, 1936). Tracing of two consecutive sclerotomes (Red and Green) based on cell density. Dotted line shows the progressive cranial shift of the sclerotome as it migrates towards the notochord. (F) Sclerotome shift model. (G) According to the sclerotome shift model, each vertebra is derived from a single somite. (H) The orientation of a grafted somite (stippled) can influence the result: even a slight deviation from the correct rostro‐caudal orientation will result in ‘like’ sclerotome cells mixing, appearing like artefactual resegmentation. (I) Schematic showing our experimental design for tracing somites. The three caudal‐most somite pairs were labelled alternately with DiI and DiO. R, rostral sclerotome; C, caudal sclerotome; DM, dermomyotome; IVD, intervertebral disc; M, muscle; NA, neural arch; NC, notochord; SCL, sclerotome;VB, vertebral body.

In 1855, Robert Remak proposed his ‘resegmentation’ (Neugliederung) model to explain this realignment, after observing that each sclerotome is subdivided into rostral and caudal halves with different cell densities, separated by an ‘intrasegmental fissure’ (Von Ebner, 1889). Remak suggested that each half‐sclerotome joins with the half‐sclerotome from the adjacent somite to form a vertebra comprising cells from two successive somites on each side of the midline (Fig. 1 A,B) (Remak, 1855). Since then numerous anatomical studies, in different amniote species, sought to address the relationship between somitic and vertebral segmentation. Some of these supported resegmentation (Von Ebner, 1889; Sensenig, 1949). However, examination of histological sections at successive developmental stages (Schauinsland, 1905; Lillie, 1936) can also lead to a different interpretation: as it migrates to surround the notochord, the entire sclerotome could shift cranially by a half‐segment relative to the corresponding dermomyotome (Fig. 1C–E). This is an alternative model for vertebral column formation: instead of a rearrangement of independent sclerotome halves, the ventral sclerotome is displaced by half a segment with respect to the myotome (Fig. 1F,G). In this model, each vertebra is derived from a single somite; both models account for how the centra are shifted relative to the myotomes. There is also a third model. Some authors point out that when the sclerotome migrates to the midline, it forms an unsegmented mass around the notochord prior to vertebral formation (Kölliker, 1861; Froriep, 1883, 1886; Baur, 1969; Verbout, 1985). It is therefore possible that vertebral boundaries are specified *de novo* from this mass (Williams, 1908; Verbout, 1976, 1985), disconnecting somitic and vertebral segmentation.

The anatomical studies upon which all of the above models are based used morphological landmarks (such as a fissure or cell density differences) as markers of sclerotome boundaries. However, morphological features are not reliable indicators of cell lineage. The first experimental studies to address this question used quail‐chick grafts to trace somite fate (Beresford, 1983; Aoyama & Asamoto, 1988; Bagnall et al. 1988; Goldstein & Kalcheim, 1992; Huang et al. 1996) and concluded that a single somite contributes to two successive vertebrae, supporting the resegmentation model. However, ‘like’ sclerotome halves (i.e. either two rostral halves or two caudal halves) have been shown to mix when grafted adjacent to each other (Stern & Keynes, 1987). This raises the possibility that even a modest deviation of the grafted somite from its correct orientation may lead to juxtaposition of ‘like’ cells, causing artificial resegmentation (Fig. 1H) (Stern, 1990). To circumvent this problem, grafts of 1.5 somites were carried out, allowing rostro‐caudal polarity to be controlled more easily (Huang et al. 2000a). The results supported the idea that each centrum, as well as the associated neural arch, is derived from two successive somites. Further support for resegmentation came from grafts of either the caudal or rostral sclerotome half: caudal half grafts contributed to the rostral part of a centrum and vice versa (Goldstein & Kalcheim, 1992; Aoyama & Asamoto, 2000). These grafting studies led to the abandonment of the other models in favour of resegmentation as the accepted model for vertebral formation in amniotes.

However, there are discrepancies between these studies regarding the contribution of each somite to the neural arches and ribs (Huang et al. 1996; Aoyama & Asamoto, 2000). Furthermore, they all rely on grafting, which may disrupt normal cell behaviour, and the extent of cell mixing may also be affected by quail‐chick differences (Bellairs et al. 1981). An alternative approach is to label somites *in situ*. This has been attempted using carbocyanine dyes (Bagnall, 1992) or retroviral transduction of a LacZ marker (Ewan & Everett, 1992). However, problems with fluorescent signal persistence after long incubation periods in the former, and the inability to contain the retrovirus in a single somite in the latter, render the results inconclusive. Peanut agglutinin, which stains the caudal sclerotome half (Stern et al. 1986; Davies et al. 1990) has also been used (Bagnall, 1989) but molecular markers are not good lineage tracers because they may be expressed by different cells at different stages. Overall, none of these studies provides conclusive proof for any of the proposed models for vertebral formation.

Here we re‐examine this issue using improved carbocyanine dyes (CellTracker CM‐DiI and SP‐DiOC_18_(3)) to label somites *in situ* and trace their contributions to the vertebral bodies and neural arches along the chick vertebral column (Fig. 1I). In contrast to previous studies (Bagnall, 1992), fluorescence persists in the vertebral column 6 days after labelling. We also test whether the relationship between somites and vertebrae varies in different body regions, which could account for discrepancies between previous studies. Such variation is suggested by a recent study using a transgenic approach to trace sclerotome fate in mouse, which found regional differences in the relative contribution of each sclerotome half to the vertebral bodies (Takahashi et al. 2013). Our results support Remak's resegmentation model but also reveal a shift between dorsal and ventral sclerotome elements in the lumbosacral region. We also clarify the somitic composition of the atlanto‐axial components. Based on these results, we propose an alternative model for amniote vertebral segmentation.

## Materials and methods

### DiI/DiO labelling

Fertile domestic fowl eggs (*Gallus gallus*; Brown Bovan Gold, Henry Stewart & Co., UK) were incubated at 38 °C in a humidified incubator and staged (Hamburger & Hamilton, 1951). Eggs were prepared as described in Stern & Holland (1993). Stock solutions of 2 mm CellTracker^™^CM‐DiI and SP‐DiOC18(3) (Molecular Probes^™^; referred to as DiI and DiO) in dimethylformamide were diluted to concentrations of 150  and 230 μM respectively, in 0.3 m sucrose containing 0.002% Tween‐20. DiI or DiO was injected into the somitocoel of the caudal‐most three somites (red/green/red from rostral to caudal) on each side of the midline (Fig. 1I) using a fine pipette pulled from a 50‐μL borosilicate capillary (Sigma) attached to an aspirator tube. Labelled embryos were incubated for a further 6 days and fixed in 4% paraformaldehyde (PFA) overnight at 4 °C, washed in phosphate buffered saline (PBS), dissected ventrally to reveal the vertebral column, sectioned using a microsurgical knife and photographed with an Olympus SZX10 microscope with epi‐fluorescence.

### Skeletal preparations and neural arch measurements

To examine the skeleton, embryos were stained with Alcian Blue and Alizarin Red S (McLeod, 1980). After staining, the vertebral column was isolated, pinned out with its lateral surface uppermost on a silicon‐based Petri dish (Sylgard, Dow Corning), and photographed using an Olympus SZX10 microscope. The angle of projection of each neural arch was measured from these images as shown in Supporting Information Fig. S1A.

### Vibratome sectioning

HH28 embryos were fixed in 4% PFA overnight at 4 °C and washed in PBS. Embryos were then eviscerated and embedded in 7% Ultrapure^™^ LMP Agarose (Invitrogen). Sagittal sections of 150–200 μm were made with a vibratome series 1000 and collected in ice‐cold PBS.

### *In situ* hybridisation

*In situ* hybridisation was carried out using a digoxigenin‐labelled *Hoxa10* cDNA probe as described (Stern, 1998). The chick *Hoxa10* plasmid was a gift from Cliff Tabin (Burke et al. 1995). For sections, the duration of Proteinase K treatment was 20 s. For whole mounts it was 20 min. Agarose was melted and removed from the sections during the pre‐hybridisation step by incubating in 70 °C hybridisation buffer for 10 min, before replacing with fresh pre‐warmed buffer. Stained sections were mounted in Glycergel® (Dako) and photographed using an Olympus VANOX‐T microscope. Embryos stained in whole‐mount were photographed using an Olympus SZX10 microscope.

## Results and Discussion

### Somite tracing supports ‘resegmentation’ in the vertebral bodies

**Figure 2:**
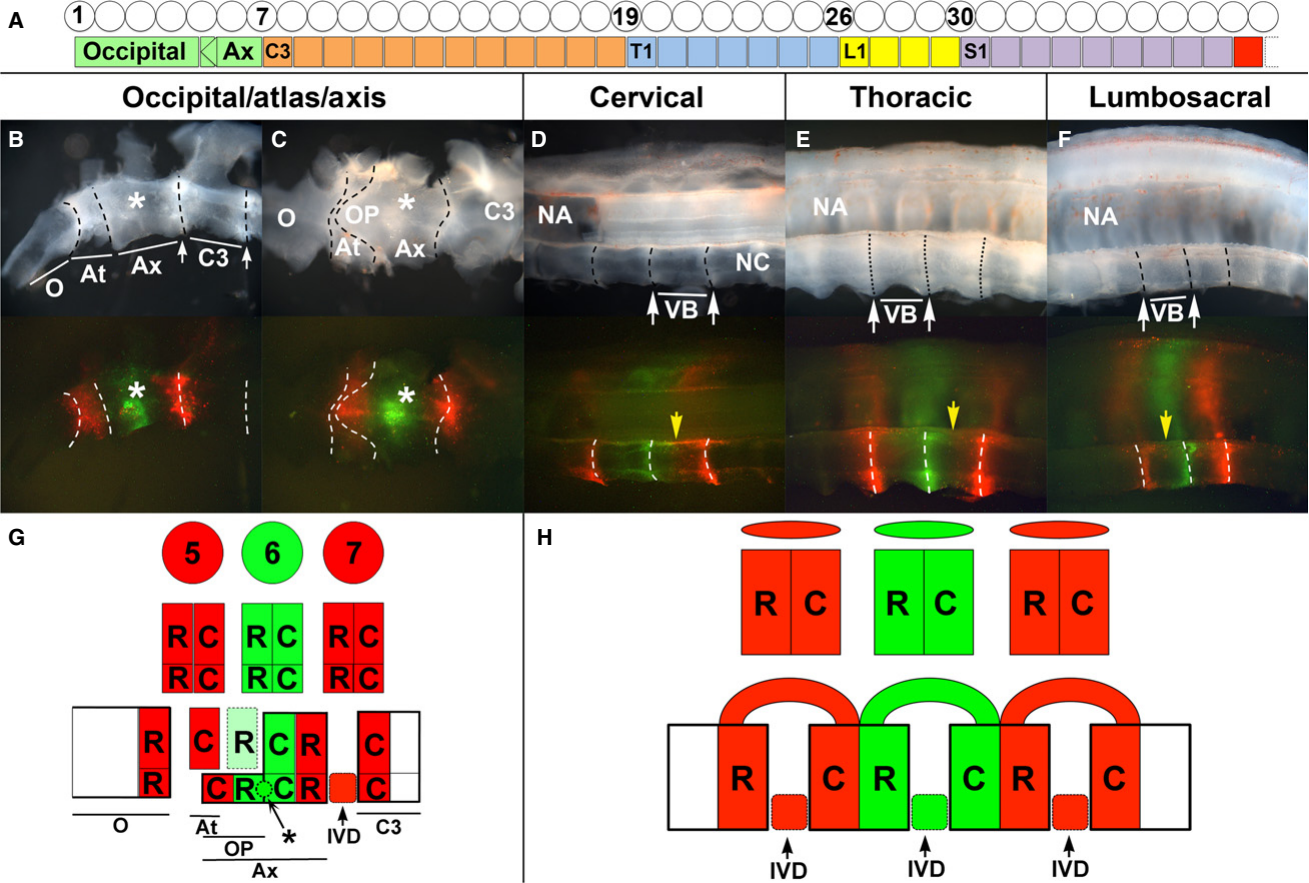
Somite fate in the vertebral column. (A) Fate of each somite (circles) in the vertebrae (squares) (Burke et al. 1995). Green = occipital, atlas and axis. Orange = cervical. Blue = thoracic. Yellow = lumbar. Purple = sacral. Red = caudal. (B–F) Somite fate in the vertebral bodies. Rostral to the left and dorsal to the top. (B,D–F) Sagittal sections through vertebral column of 8‐day‐old embryos after labelling somites. Bright field images (above) show the vertebral elements; below, somite contributions to the vertebrae (DiI red, DiO green). (C) Dorsal view of the vertebral bodies in (B). The position of the intervertebral disks is marked with dotted lines. In each panel, a vertebral body (VB) is labelled and its adjacent intervertebral disks are indicated (white arrows). (G) Contribution of somites‐5–7 to the occipital‐atlas‐axis complex. (H) Fate of sclerotome in the centra in other regions of the vertebral column. At, atlas;, Ax, axis; O, occipital; OP, odontoid process; Yellow arrow, intersomitic boundary; other labelling and shapes as in Fig. 1.

To trace the fate of somite cells along the head‐tail axis, we used DiI (red) and DiO (green) to label three consecutive pairs of somites on each side of the embryo in a red‐green‐red pattern (Fig. 1I). After incubation to Hamburger & Hamilton (1951) (HH) stage 29–33 the embryos were sectioned sagittally through the centre of the vertebral column and examined by fluorescence microscopy. This was carried out in each region of the vertebral column (Fig. 2A).

Supporting Information Table S1 summarises the results. In the cervical (*n* = 3), thoracic (*n* = 4) and lumbosacral regions (*n* = 4), a single vertebral body (centrum) comprised cells from two successive somites (Fig. 2D–F,H), with the boundary between red‐ and green‐labelled cells located in the middle of the vertebral body (Fig. 2D–F,H, yellow arrow). Cells from a single somite were detected in the annulus fibrosus of the intervertebral disc and in approximately half of the vertebral bodies rostral and caudal to it. These results support the resegmentation model.

The boundary marking the contribution of two adjacent somites was always sharp, with no discernible mixing of labelled cells 6 days later (Fig. 2D–F, yellow arrows). This is consistent with the properties of rostral and caudal sclerotome halves, which form a boundary when placed in close proximity (Stern & Keynes, 1987). These results confirm that this property is strictly maintained even after the extensive migration and proliferation that accompanies vertebral development. Stronger labelling was always observed in the intervertebral discs compared with the vertebral cartilages, suggesting either that these cells divide less frequently or that injection of the dye into the somitocoel labels these cells (reported to give rise to the annulus fibrosus of the intervertebral disc; Huang et al. 1996) more intensely. Grafting experiments showed that somitocoel cells are not committed to an intervertebral disc fate (Senthinathan et al. 2012). The resegmentation mechanism accounts for the final intervertebral position of these cells, where they could receive instructive signals to become annulus fibrosus.

### Somite contribution to the occipital region, atlas and axis

The morphology of the rostral‐most vertebrae is distinctive. The atlas (C1) sits behind the occipital region of the skull, forming the atlanto‐occipital joint, which allows flexion and extension of the head. The atlas has a ring‐like shape, through which the odontoid process (OP; also known as the dens) projects from the rostral face of the bulkier axis (C2) behind. Together, the atlas and axis form the atlanto‐axial joint, which allows head rotation. As the projection of the OP is not obvious in sagittal sections (Fig. 2B), we removed the surrounding soft tissues and imaged it from the dorsal side (Fig. 2C) before sectioning (Fig. 2B) to examine this region more clearly. Cells from somite‐5 (red) were found in the caudal occipital cartilage, the entire atlas and the rostral tip of the OP, which is fused to the axis. Cells from somite‐6 (green) were found in the rostral portion of the axis body and the base of the OP. Somite‐7 (red) contributed to the caudal portion of the axis body, the disc between the axis and C3, and the rostral portion of the body of C3.

This apparent rearrangement of segments becomes clearer when the ventral (future vertebral bodies) and dorsal (future neural arches) aspects of the sclerotome are considered as separate entities (Fig. 2G). The rostral half of somite‐5 fuses with the occipital region of the skull. The atlas comprises the dorsal part of the caudal half of somite‐5, the ventral part of which fuses to that of somite‐6 to form the OP. The OP fuses to the rostral face of the axis, which comprises somites 6 and 7. Therefore, the atlas is the only vertebra to derive entirely from a single somite, whereas the axis (including the OP) receives contributions from three somites.

Our results are in agreement with classical embryological studies, which suggested that the OP is the ‘missing’ vertebral body of the atlas in birds (Hayek, 1924; De Beer & Barrington, 1934; De Beer, 1937). These studies also concluded that the OP and atlas form from the caudal half of the first ‘trunk’ sclerotome, the rostral half of which contributes to the caudal‐most part of the occipital bone. Our findings also support a previous study using quail‐chick grafts to trace the occipital/rostral cervical somites (Huang et al. 2000b), with one exception: we found no evidence of a contribution of somite‐6 to the caudal part of the atlas, therefore its fate remains unclear. Strong DiO fluorescence similar to that seen in the intervertebral discs was present in a small area at the base of the OP (Fig. 2B,C; white star). As this sits at the boundary between rostral and caudal halves of somite‐6, it probably represents a vestigial disc, the development of which was suppressed by fusion of the OP to the axis (Fig. 2G, dashed circle indicated by asterisk).

### Somite tracing supports ‘resegmentation’ in the neural arches and regional variation in the tilt of the intersomitic boundaries

**Figure 3:**
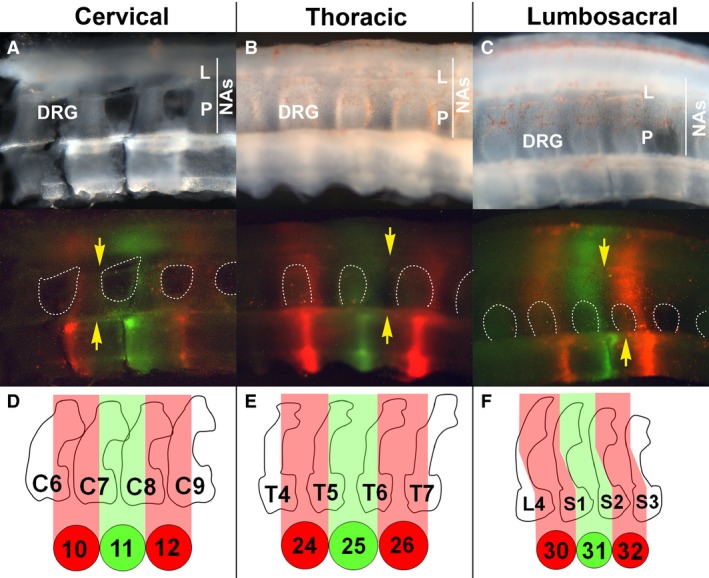
Somite fate in the neural arches. (A–C) Sagittal sections through the vertebral column of 8‐day‐old embryos after labelling somites with DiI and DiO. In the fluorescent images, the dorsal root ganglia are outlined with dashed white lines. DRG, dorsal root ganglion; other labelling as in Fig. 1 and 2 (D–F) Relationship between the intersomitic boundary and the neural arch tilt in each region, drawn from a skeletal preparation of an HH32–33 embryo. Note that in (A) the neural tube and DRGs have been removed.

The same approach was used to examine the contribution of somites to the neural arches. Each neural arch has two components. The pedicle projects dorsally from the vertebral body rostral to the dorsal root ganglion (DRG) of the spinal nerve. The lamina extends from the pedicle over the dorsal neural tube, joining the neural arches on either side of the midline. The somite contribution to the pedicle and the ventral part of the lamina could be analysed (Fig. 3A–C) in the cervical (*n* = 3), thoracic (*n* = 4) and lumbosacral (*n* = 4) regions after sagittal sectioning. For both components, each neural arch contains labelled cells from two successive somites (Fig. 3A–C). The boundary between red‐ and green‐labelled somites in the neural arch was aligned with the boundary in the middle of the vertebral body, extending dorsally through the caudal limit of each pedicle (the rostral limit of the DRG) and bisecting the lamina above (Fig. 3A–C, yellow arrows). Thus, the pedicle appears to be derived almost entirely of cells from the more anteriorly labelled somite (presumably its caudal half), with only a small, if any, contribution to the back of the pedicle made by the next‐posterior somite. Our results are consistent with previous whole and half‐somite grafting studies (Bagnall et al. 1988; Huang et al. 1996, 2000a; Aoyama & Asamoto, 2000). They also fit with the finding of Goldstein & Kalcheim (1992) that only the caudal sclerotome can give rise to the pedicle.

Although the fate of somites in the neural arches was similar in all three regions, the orientation of the red–green boundary was variable. In the cervical and thoracic regions the boundary runs vertically from the centrum to the dorsal neural arch, showing that cells from a single somite migrate to a similar level along the head–tail axis regardless of whether they give rise to ventral or dorsal structures (Fig. 3A,B). However, in the lumbosacral region the red‐green boundary tilts rostrally, suggesting that somite cells shift as they contribute to progressively more dorsal structures within the same segment (Fig. 3C). These results suggest that in the lumbosacral region, a mechanism exists that combines features of the first two models presented above: true resegmentation along with a ‘shift’ between dorsal and ventral cells derived from the same sclerotome.

To investigate how this ‘tilt’ in the intersomitic boundary relates to neural arch morphology, we examined skeletal preparations of 8‐day‐old embryos (Fig. S1A; *n* = 6) and measured the tilt of the pedicles relative to the horizontal axis of the vertebral bodies for the vertebrae corresponding to those analysed in Fig. 3A–C (Fig. S1B). On average, the pedicles in the lumbosacral region projected at a more acute angle (M = 72°, SD = 6°) compared with those in the cervical (M = 91°, SD = 2°) or thoracic (M = 91°, SD = 3°) region, mirroring the regional variation in the ‘tilt’ of the intersomitic boundary (Fig. 3D–F). Thus, the intersomitic boundary follows the angle of the pedicle, suggesting that the relationship between somite and vertebral segmentation is maintained along the body axis, and the morphology of the neural arch is related to the position of the sclerotome at the midline.

### The boundary of *Hox* gene expression in the vertebrae further supports resegmentation

Surprisingly, the precise position of the boundaries of *Hox* gene expression have not been mapped in relation to the somites and vertebral column elements, so it is unclear whether the most rostral limit of expression corresponds to the border between somites or between vertebrae. We examined this for *Hoxa10* and found that at somite stages the expression domain ends rostrally at a somite boundary and later, when the skeletal elements form, the boundary is found in the middle of the neural arch and corresponding vertebral body (Supporting Information Fig. S2). This is consistent with resegmentation.

### Resegmentation and shifting sclerotomes

**Figure 4:**
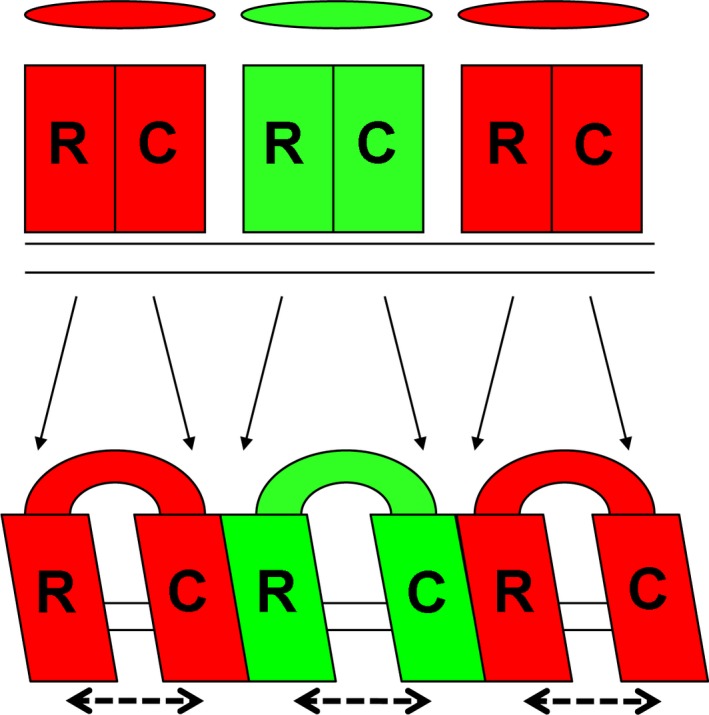
The resegmentation‐shift model: resegmentation of the sclerotome is accompanied by a shift of variable extent along the axis (dashed arrows). Shapes and labels as in Fig. 1.

Here we demonstrate, without grafting, that the vertebral bodies and neural arches form by resegmentation of the sclerotome, in agreement with the Neugliederung model proposed over 150 years ago (Remak, 1855). In addition, our results suggest that in the lumbosacral region, dorsal and ventral sclerotome cells from the same somite shift relative to one another, leading to a rostral ‘tilt’ of the intersomitic boundary that correlates with the slant of the neural arch pedicle. We propose a ‘resegmentation‐shift’ model, in which the final vertebral pattern is established by resegmentation of the sclerotome plus a shift, which varies along the head–tail axis (Fig. 4).

## Supporting information


**Fig. S1.** Measurements of the ‘tilt’ of the neural arch.

**Fig. S2**. The boundary of Hox gene expression corresponds initially to a somite boundary and later to the middle of a skeletal element.

**Table S1.** Summary of experimental embryos labelled and sectioned. Embryos used for Figs 2 and 3 are highlighted in bold.
