# Supplementary material for: A resegmentation‐shift model for vertebral patterning
Source: J Anat. 2016 Sep 1;230(2):290–6. doi: 10.1111/joa.12540 (PMC5244455; doi:10.1111/joa.12540)
Supplement: Supplementary file 3 — Table S1. Summary of experimental embryos labelled and sectioned. Embryos used for Figs 2 and 3 are highlighted in bold. [file JOA-230-290-s003.docx]

**Supplementary Table S1**

| **Embryo** | **Axial region** | **Somite no. labelled** | | | **Vertebral element analysed** | |
| --- | --- | --- | --- | --- | --- | --- |
|  |  | **DiI** | **DiO** | **DiI** | **Vertebral bodies** | **Neural arches** |
| 090414(1) | Axis/Atlas | 5 | 6 | 7 | X | - |
| 090414(2) | Axis/Atlas | 5 | 6 | 7 | X | - |
| **281112(4)** | **Axis/Atlas** | **5** | **6** | **7** | **X** | - |
| **141112(4)** | **Cervical** | **9** | **10** | **11** | **X** | - |
| 210313(1) | Cervical | 10 | 11 | 12 | X | - |
| 210313(3) | Cervical | 10 | 11 | 12 | X | - |
| **190613(2)** | **Cervical** | **10** | **11** | **12** | **-** | **X** |
| 261113(1) | Cervical | 10 | 11 | 12 | - | X |
| 261113(2) | Cervical | 11 | 12 | 13 | - | X |
| 041013(1) | Thoracic | 24 | 25 | 26 | X | X |
| **041013(3)** | **Thoracic** | **24** | **25** | **26** | **X** | **X** |
| 220313(3) | Thoracic | 19 | 20 | 21 | X | - |
| 220313(1) | Thoracic | 19 | 20 | 21 | X | - |
| 141013(3) | Lumbar | 27 | 28 | 29 | X | X |
| 141013(1) | Lumbar/Sacral | 29 | 30 | 31 | X | X |
| 190413(2) | Lumbar | 27 | 28 | 29 | X | X |
| **141013(4)** | **Sacral** | **30** | **31** | **32** | **X** | **X** |

**Table S1.** Summary of experimental embryos labelled and sectioned. Embryos used for figures 2 and 3 are highlighted in bold.
